# Report of the Dean of the Faculty of the Medical Department of the Iowa State University to the Superintendent of Public Instruction

**Published:** 1855-09

**Authors:** 


OF THE

### Dean of the Faculty of the Medical Department

OF THE

### IOWA STATE UNIVERSITY,

TO THE

### SUPERINTENDENT OF PUBLIC INSTRUCTION.

NOVEMBER 30, 1854.

*Keokuk, Iowa*, Jan. 15th, 1854.

*Gentlemen of the Senate*
*and House of Representatives:*

By reference to the report of the Superintendent of Public Instruction, presented to your honorable body the present session, it will be seen that the report of this institution has been omitted. Astonished at this, I feel myself called upon to state the facts in the case. Mr. Eads well knows that the report referred to was handed him in person by our representative, the Hon. R. P. Creel, on the evening of the 2nd ult. His report is dated Dec. the 4th, two days after the receipt by him of my report, and several days previous to its presentation to your honorable body. The design of the Superintendent in suppressing the report, is best known to himself, and is just cause of much suspicion. Such procedure by a public functionary in withholding from the people's representatives facts which it is their legitimate right to know, is a proceeding too high-handed to pass unnoticed.

In justice to myself, and in behalf of the Medical Faculty of a state institution, I have thought it my duty to deny the truth of the statement of the Superintendent, and as a recent demand of the House, of the Superintendent, for a copy of my report, cannot be complied with, owing to his absence, I have thought it proper to present each member of your honorable body a copy which has been taken from the original manuscript, and will not vary in meaning from the one already in the hands of the Superintendent.

With much esteem

I am very respectfully yours,

J. C. HUGHES,

Dean Medical Faculty Iowa University.

To the Hon. JAMES D. EADS,

*Superintendent Pub. Instruction:*

Sir:

The regulations established by our State Legislature in relation to the different departments of the Iowa University, require me, as Dean of this the Medical Department, to submit to its honorable body, through you, a report of the Financial and Scientific condition of said department.

Since the last report presented by the former Dean, John F. Sanford, (which report was not approved by the Faculty,) to the Hon. Thomas H. Benton, Jr., and through him to the last General Assembly, much of interest has transpired in this department.

From the time of the location of the institution in this place, and up to the date of that report, our classes were small, numbering the first session seventeen matriculants, the second session nineteen, and the third session, being the time at which the former report was made, but fifteen students were present. The unusually small attendance at that time, and the embarrassments under which the institution labored, were most discouraging to the Faculty.

The General Assembly, at its session of 1850, appropriated to this department of the University the sum of five thousand dollars, from the proceeds of the sales of the saline lands. This sum was made available, as will be seen by reference to the report of the former Dean, by receiving from the city of Keokuk her bonds, giving the state appropriation in pledge for their payment. This amount was set apart for special purposes, as wiil be seen by referring to pages 5 and 6 of the Superintendent's report of 1852. A part of the money having been received upon two of the bonds, from Messrs. Bangs & Bros. of New York, as may be seen by reference to the last report, (see page 9,) the application of this fund was entrusted by resolution of the Faculty to the then acting Dean, John F. Sanford, and was reported by him to have been expended as follows:

(See page 10 of Superintendent's report, 1852.)

*Tabular Exhibit.*

| | |
|---|---|
| Amount paid by Messrs. Bangs & Bros. | $1600 00 |

The account of the expenditure of this money was kept by the Hon. Mayor of this city, with whom the principal part was deposited, and to whom vouchers for the whole were exhibited and approved. The following is an exact copy of the account as rendered by him:

| | |
|---|---|
| Amount paid R. P. Gray, contractor on College building, - - - - - - | $761 00 |
| Boatman for Lumber, - - - - - - | 15 00 |
| J. A. Graham, cash advances, - - - | 81 25 |
| Shepard & Volentine, - - - - - - | 60 00 |
| Cleghorn & Harrison, - - - - - - | 50 61 |
| Edward Tarbell, - - - - - - - - | 4 60 |
| Benjamin Pike, Jr., N. Y., Chemical Apparatus, | 127 00 |
| do do Microscope, - | 148 00 |
| Joseph Brano, Wax Specimens, - - - | 54 00 |
| C. E. Isaacs, - - - - - - - - | 125 00 |
| J. L. Hatch, (Agent,) - - - - - - | 8 37 |
| Bill of expenses incurred on trip from Keokuk to New York and return, as per bill of particulars, giving the items, &c., - - | 132 25 |
| Drayage, Porterage, &c., - - - - - | 4 60 |
| | $1672 89 |

Contrary to the provisions in the laws of the College, the Dean at the time selected as temporary Treasurer the Hon. Mayor of this City, with special reference to this fund. After the return of the Dean from New York City, where he had been to purchase appliances, the disbursement of the fund was reported to the said temporary Treasurer, to whom vouchers were exhibited for the payment of the several sums as expended. The said Treasurer saw these vouchers, as is shown by the following certificate:

(Copy of Certificate—see page 10 former report.)

I hereby certify that John F. Sanford, Dean of the Medical Institution of the City of Keokuk, Iowa, produced to me full and satisfactory vouchers for the payments made by him as Dean as aforesaid, to the amount stated, say 1672 dollars and 89 cents, which will overpay the amount which was deposited in my hands as Treasurer, the sum stated, seventy-two dollars and eighty-nine cents.

(Signed) JOHN A. GRAHAM.

The amount overpaid, $72 89, is again charged to the College, being included in John F. Sanford's bill, as you may see by reference to 23d line on page 12 of former report.

After this report was made, circumstances transpired which led some members of the Faculty to suspect a misapplication of the funds by the Dean, and investigations were made which resulted in the following disclosures:

For the article of Microscope, purchased from Benjamin Pike, Jr., of New York, he reports to have paid $148 00, and presented a voucher to this effect, whereas the duplicate of Mr. Pike's bill, on file in this office, shows that the cost of the instrument was but

$48 00. Here is a deficiency which has not been explained. Also the bill of C. E. Isaacs he reports to have been $125 00, when a letter on file in this office from Dr. Isaacs states that the amount paid was but $25 00. Thus the institution has lost $200 which was appropriated by the State for its benefit, as shown by these exhibits, and as soon as he (the Dean) was informed by the Faculty that these documents were in their possession, he resigned his connection with the institution, leaving the chair of Surgery vacant.

A portion of the bonds upon which no payment had been made, was deposited with the house of Cox & Shelley, of this city, who met the indebtedness of the institution to the amount in their hands, the items of which may be found on the 11th page the former report. The amount of indebtedness of the institution at the time said report was made, will be seen by reference to page 12 to have been as follows:

| | |
|---|---|
| Amount paid by Messrs. Perkins & Pitmann to L. J. Swart, interest to date, - - - | 33 56 |
| Paid by C. Garber & Co. with interest to date, - | 182 50 |
| Paid by J. E. Burke, - - - - - - | 61 83 |
| Balance due Messrs. Curtis & Gilmore, - - | 78 51 |
| Amount due Mr. Dewey, (about,) - - - | 75 00 |
| Paid by Mr. Anderson to Swart, - - - | 41 38 |
| Paid by Bridgman & Reed, - - - - | 125 00 |
| J. F. Sanford for money paid for Chemical Apparatus and other purposes, - - - | 297 00 |
| Amounting in all to - - - - | $894 78 |

This amount, $894 78, is represented in the report as being the total indebtedness of the institution at the date of statement, but the Faculty have since canceled several claims contracted previous to that date.

The amount of indebtedness noted, $894 78, has been canceled by individual members of the Faculty, with the exception of two items, viz: that of Bridgman & Reid, $125 00, and that of John F. Sanford, $297 00 in all, for which no definite arrangement has been made.

Besides the two hundred dollars specified above, the former Dean still holds a balance due from Messrs. Bangs & Bros. as will appear on page 11 of his report; also Surgical appliances belonging to the institution.

Although laboring under these embarassments, yet feeling that the interests of the profession of our young and flourishing State demanded a renewed effort on the part of the Faculty, to sustain this department of the University, it was resolved by the remaining members to supply vacancies, and use every effort more fully to sustain it.

The Faculty was then organized by the appointment of

D. L. McGUGIN, M. D.,
Professor of Physiology, Pathology and Microscopy.

FREEMAN KNOWLES, M. D.,
Professor of Theory and Practice of Medicine.

J. C. HUGHES, M. D.,
Professor of Surgery and Dean of Faculty.

J. E. SANBORN, M. D.,
Professor of Chemistry and Meteria Medicia.

E. R. FORD, M. D.,
Professor of Obstetrics and Diseases of Women and Children.

E. A. ARNOLD, M. D.,
Professor of Anatomy.

P. VAN PATTEN, M. D.,
Demonstrator of Anatomy.

This reorganization has resulted in making this department of the University more decidedly a State institution, and as such a lively interest has been awakened in the public mind in its favor, and the profession looks upon it as dispensing great good and as contributing to its best interests.

The chair of theory and practice becoming vacant before the opening of the session of 1853-'54, Freeman Knowles, M. D., who has been a successful practitioner in this State for the last thirteen years, was appointed to fill the vacancy.

Before the Faculty were enabled, by proceed of sales of saline lands to meet the claims due the City of Keokuk on her bonds issued for our benefit, interest had accumulated on said bonds to amount of some six hundred dollars. The City Council, knowing the many difficulties under which the institution was laboring, have in their wisdom and generosity released the College from the indebtedness.

During the session of 1852-'53, at which time the last report was made to the Hon. Thos. H. Benton, Jr., by the former Dean, our class numbered, as has been stated, but 15 students. After the reorganization of the Faculty and during the session of 1853-'54, the nember of matriculants amounted to 41, while the whole number in actual attendance upon the lectures was about 50. During the present session, the number of students has been largely increased beyond that of last winter, the number of matriculants being already between 65 and 70, and will reach 75 before the close of the session.

This rapid increase seems to be sufficient evidence of the estimation in which the institution is held by its numerous friends throughout the State, and also of the energy of the Faculty as well as their ability to instruct. We may add that the number of graduates at the close of the session of 1852–'53 was only 6! The number that graduated at the close of the last course of lectures, 1853–'54 was 13. While already the number of candidates for graduation at the close of the present course is 20.

It has become my duty to mention these facts as evidence of the increased prosperity and usefulness of the medical department. It is also a pleasure to me to record the uniformly harmonious and united efforts of the Faculty, during two years of difficulties and embarassment. Nor must I omit to mention the courtesy and enthusiasm of the students, as evinced by their gentlemanly deportment, and their punctual and attentive presence at lectures.

While the efforts of the Faculty have been thus earnestly directed to the extension of the influence of the institution at home, the interests of this department have not been overlooked in the visits which several of the professors have made to the prominent Medical Colleges and Hospitals of the East during the last two years.

In the spring and summer of 1853, the Professor of Surgery spent some four months in the Cities of Baltimore, Philadelphia and New York, diligently employed in the cultivation of his department; at this visit, he was also the delegate of the College and the State, at the annual meeting of the American Medical Association.

During the last season, Prof. Sanborn visited New York and Boston, paying more particular attention to recent progress in chemical science, while Professors Ford and McGugin have each visited different portions of the the East and the prominent medical schools.

During the month of August, 1853, since the last report to your predecessor, the first number of the Iowa Medical Journal was issued, a work designed to be the exponent of the Medical literature and character of this portion of the country, conducted by the Faculty, and identified with the Medical College. This journal was originated because there was none in this state. Its success has exceeded our most sanguine expectations. It has already entered upon its second volume, much enlarged and improved. It exercises a healthful moral and professional influence in its sphere, and its position and rapid improvement are a sufficient proof of the estimation in which it is held by its numerous patrons and contributors.

The Anatomical and Medical Museum has shared largely in the improvements which have been made in the various departments of the institution. A large number of wet and dry preparations, representing numerous important points in general, special, and pathological Anatomy, are on exhibition for reference and observation,

A very numerous collection of Botanical plates, placed in the institution by one of its Faculty, is an efficient aid in the lectures upon Materia Medica. A number of valuable works have been furnished from various sources, by exchange with the Iowa Medical Journal and by direct donations from members of Congress and the Smithsonian Institute, which go to form a very respectable nucleus for what we hope may in time be a creditable Library.

After having presented a condensed statement of the Scientific and Financial condition of the institution, we would in conclusion ask a continuance of that fostering care and favor of the Legislature which has heretofore been extended to it. The Faculty in turn can but promise a faithful discharge of our responsible and arduous duties connected with this most important scientific enterprise in the state, which we ardently hope and believe, under the present government and management, will maintain its elevated and promising condition in the estimation of the American Medical profession and a generous public.

With great respect
I am, dear sir,
Yours,
J. C. HUGHES,
Dean Medical Faculty Iowa University.

Keokuk, Nov. 30th, 1854.

